# Quantitative Proteomics via High Resolution MS Quantification: Capabilities and Limitations

**DOI:** 10.1155/2013/674282

**Published:** 2013-04-23

**Authors:** Richard E. Higgs, Jon P. Butler, Bomie Han, Michael D. Knierman

**Affiliations:** ^1^Global Discovery and Development Statistics, Lilly Research Laboratories, Indianapolis, IN 46285, USA; ^2^Lilly Corporate Center, DC 0720, Indianapolis, IN 46285, USA; ^3^Translational Sciences, Lilly Research Laboratories, Indianapolis, IN 46285, USA

## Abstract

Recent improvements in the mass accuracy and resolution of mass spectrometers have led to renewed interest in label-free quantification using data from the primary mass spectrum (MS1) acquired from data-dependent proteomics experiments. The capacity for higher specificity quantification of peptides from samples enriched for proteins of biological interest offers distinct advantages for hypothesis generating experiments relative to immunoassay detection methods or prespecified peptide ions measured by multiple reaction monitoring (MRM) approaches. Here we describe an evaluation of different methods to post-process peptide level quantification information to support protein level inference. We characterize the methods by examining their ability to recover a known dilution of a standard protein in background matrices of varying complexity. Additionally, the MS1 quantification results are compared to a standard, targeted, MRM approach on the same samples under equivalent instrument conditions. We show the existence of multiple peptides with MS1 quantification sensitivity similar to the best MRM peptides for each of the background matrices studied. Based on these results we provide recommendations on preferred approaches to leveraging quantitative measurements of multiple peptides to improve protein level inference.

## 1. Introduction

The ability to identify and quantify hundreds or even thousands of peptides in a single data dependent LC/MS analysis of a proteolytic digest from a complex mixture such as serum or plasma established mass spectrometry-based proteomics as a tool of choice for biomarker discovery research. These are the ultimate hypothesis-neutral experiments that have the promise of finding solutions for major gaps in drug discovery and other biological inquiries. In the past several years, remarkable advances have been made to better support these efforts on multiple fronts, including sample preparation, instrumentation, and data processing. Proteins and peptides that are part of specific biological pathways tend to exist in low nanomolar or lower concentrations, while the abundant proteins in human serum reach concentrations in the hundreds of micromolar range. For example, a typical concentration of apolipoprotein A1 in human serum is around 30 *μ*M while that of ghrelin is around 100 pM. This five-plus log difference in dynamic range is well beyond the current capabilities of analytical mass spectrometry methods. In order to measure peptides of such low abundance, enrichment of the target analyte using antibodies or other methods before analysis by mass spectrometry is required. 

When mass spectrometric measurements require prior enrichment, the advantage mass spectrometry provides over immunoassay-based detection methods might be in question. We propose that for hypothesis generation applications, there are multiple reasons to employ a broader proteomics approach. Immunoassay-based detection methods cannot distinguish many post-translational modifications or can be completely blind to any unexpected modifications on the target proteins or peptides, whereas a nonbiased mass spectrometry-based proteomics approach with prior enrichment has the potential to detect and quantify multiple different forms of the target proteins and their interacting partners. The ability to detect and quantify multiple different forms of the target protein(s) is particularly important in the early stages of biomarker hypothesis generation and drug development (e.g., chemiproteomics, detailed profiling of post-translational modifications or proteolytic cleavage events using immunoprecipitation mass spectrometry, quantitative profiling of protein-protein interactions, and quantitative analysis of enriched classes of proteins). While methods for some of the known modifications or interacting proteins can be incorporated into multiple reaction monitoring (MRM) based methods, these methods will be blind to unexpected modifications or interacting proteins without prior specification. In some cases, it is also practically impossible to pre-specify MRM transitions for a large set of known modifications. Combinations of acetylation, methylation, phosphorylation, ubiquitination, and citrullination on histone proteins is a good example that is practically impossible to approach using MRM methods but has been an approachable problem with a targeted proteomics method.

Label-free quantification of MS1 spectra has the most flexibility among the different mass spectrometry based proteomics methods that have been developed. In this method, data-dependent MS2 spectra provide identification of peptides while the peak height or area of the extracted ion chromatogram (XIC) of the MS1 for the peptide parent ion provides quantitative information. Label-free MS1 quantification was originally described by Higgs et al. [1] and numerous variations of the approach have subsequently been described, including MSQuant [2], MaxQuant [3], MASIC [4], Census [5], Spectrum Mill [6], SuperHirn [7], and Skyline [8]. Spectral counting methods to quantify relative protein levels have also been described but suffer from a small quantitative range, are not able to quantify at the peptide level, and while easier to implement, are not generally competitive with the MS1 quantification methods [9]. Several of the MS1 quantification methods, including the method described in this report, are also compatible with stable isotope labeling by amino acids in cell culture (SILAC) for experimental designs that require a labeled internal standard to reduce variability or metabolic (i.e., pulse chase) experiments [10]. Isobaric labeling approaches, like iTRAQ, where quantification is done using MS2 fragment intensities have been used for the applications we describe though their cost, complexity, and lack of apparent advantages relative to MS1 quantification in our experience and in comparative studies have led many groups to focus on MS1 quantification [11, 12].

When protein samples are digested with a protease and subject to a proteomics mass spectrometric analysis, most proteins generate multiple peptides, and the question of how best to combine the information from multiple peptides to infer changes in concentration at the protein level between different treatment groups naturally arises. An arithmetic average of peak areas will be dominated by high intensity peptides, whereas a geometric mean of the peak areas (or average value of the log of the peak intensities) will result in treating high intensity peptides (often with high signal to noise ratio) and low intensity peptides (often with low signal to noise ratio) more equally. Chang et al. have proposed a linear mixed model based approach as a principled approach to modeling multiple peptide measurements from a protein [13], but other approaches need to be explored to best utilize the information-rich proteomics data. For example, even with prior enrichment of the target protein(s), most samples carry different degrees of nonspecific proteins or contamination. Under these circumstances, it is common to find peptides of interest that are heavily contaminated by interferences and, thus, provide misleading information. Therefore, any method of quantification at the protein level from the digested peptides needs to systematically address how to handle signals of varying quality from the corresponding peptides. 

 The applications of interest motivating this work include discovery mode and hypothesis generation experiments in which some form of sample preparation is done in order to enrich for the protein(s) of interest. This enrichment step is critical to identifying and quantifying the lower concentration proteins that are generally of most biological interest. Specific examples of the applications we are addressing include: characterization of post-translational modifications, *in vivo* proteolytic cleavage events, and co-precipitating proteins using immunoprecipitation; chemiproteomics applications to identify which proteins specifically interact with small molecules; and enrichment of specific classes of proteins (e.g., phospho tyrosine, acetylated, etc.) for profiling changes tied to the biology under investigation.

Clearly, inference should always be done at the peptide level in order to screen for biological events specific to a peptide (e.g., post-translational modification). However, how to best combine information from multiple peptide measurements from a protein in order to improve protein-level inference remains an open question. In this report we propose and characterize several methods to summarize quantitative peptide information for inference at the protein level. We characterize these methods using serial dilutions of a standard protein digest in background matrices of varying complexity. Additionally, the relative strengths and weaknesses of discovery-mode MS1 quantification are compared to MRMs on the same sample set and similar analytical systems. From these analyses we propose a relatively simple yet powerful method for mass spectrometry proteomics quantification that can be implemented in a relatively straightforward manner.

## 2. Materials and Methods

### 2.1. Materials

Six bovine protein tryptic digest exponential mix (PTD/00001/64), which is a mixture of tryptic digests from six bovine proteins in descending log concentrations (“sixlog mix”) and yeast enolase tryptic digest (PTD/00001/46) were purchased from Bruker Michrom. The Universal Proteomics Standard Set (UPS1) was purchased from Sigma Aldrich. Modified sequencing-grade trypsin was purchased from Promega. One milliliter ampoules of trifluoroacetic acid (TFA) were purchased from Thermo Pierce. Water, 0.1% formic acid in water, and acetonitrile (ACN) was purchased from Thermo Fisher, optima grade. Ammonium bicarbonate, sodium carbonate, urea, iodoethanol, and triethylphosphine were analytical grade. The HeLa cell lysate was produced in house from suspension culture of the cells. 

### 2.2. Sample Preparation

One vial of yeast enolase digest containing 500 picomoles was dissolved in 33 *μ*L of 50% acetonitrile, 0.1% trifluoroacetic acid to make a stock solution of 15 picomoles per microliter concentration. For each background matrix tested, the yeast enolase stock was diluted tenfold in the background matrix by adding 5 *μ*L of the enolase stock solution to 45 *μ*L of the background solution to make the dilution stock, which was subsequently used to generate dilution series in each matrix by making 3-fold serial dilutions in each matrix.

### 2.3. Preparation of Background Matrices

For sixlog mix, one vial was dissolved in 50 *μ*L of 50% ACN, 0.1% TFA, and 20 *μ*L of this solution was diluted with 1980 *μ*L of 0.1% TFA in water to yield a dilution matrix solution containing 100 femtomoles of total protein per *μ*L. 

For the UPS1 matrix, one vial with 5 picomoles each of 48 human proteins with a total protein content of 6 *μ*g was reconstituted with 10 *μ*L of 8 M urea, 50 mM sodium carbonate, 1% iodoethanol, and 0.25% triethylphosphine. The solution was incubated at 37°C for 1 hour and taken to dryness in a vacuum concentrator. The dried contents were dissolved initially in 10 *μ*L of 50 mM ammonium bicarbonate (pH 9) before an additional 87 *μ*L of 50 mM ammonium bicarbonate were added to dilute the urea concentration before addition of trypsin (60 ng of modified Promega trypsin in 3 *μ*L of 50 mM ammonium bicarbonate) and incubation at 37°C for 48 hrs. The digested sample was concentrated and desalted by passing through 3 Ziptips (Millipore) in series, with 3 passages each time. Each tip was washed ten times with 200 *μ*L of 0.1% TFA in water before eluting the peptides with 10 *μ*L of 50% acetonitrile by slowly aspirating and dispensing 5 times. The eluted materials were combined and diluted to 1 mL with 0.1% TFA in water. 

For preparation of the HeLa cell lysate matrix, 10 *μ*L of the cell lysate with total protein concentration of 6 mg/mL was mixed with 90 *μ*L of 8 M urea, 50 mM sodium carbonate (pH 11), 1% iodoethanol, 0.25% triethylphosphine, incubated at 37°C for 1 hour and dried in a vacuum concentrator. The dried sample was first dissolved in 100 *μ*L of 50 mM ammonium bicarbonate before addition of 870 *μ*L of 50 mM ammonium bicarbonate and 30 uL of trypsin (600 ng Trypsin Gold from Promega in the same buffer) and incubation at 37°C for 48 hrs. The sample was concentrated and desalted with a Sep-Pak tC18 Plus Light cartridge (Waters, Cat. No. WAT036805). The sample was passed over a prewetted cartridge twice with a 1 mL all-plastic syringe, washed two times with 1 mL of 0.1% TFA in water, then eluted from the cartridge with three 100 *μ*L volumes of 50% acetonitrile, 0.1% TFA in water. The eluted material was dried and redissolved in 50 *μ*L of 50% acetonitrile, 0.1% TFA in water and diluted to 2 mL with 0.1% TFA in water. 

### 2.4. Data-Dependent LC/MS

A Thermo Easy nLC capillary HPLC system was used in line with a Thermo LTQ-Orbitrap Velos mass spectrometer for data acquisition. Solvent A was 0.1% formic acid in water and solvent B was 0.1% formic acid in acetonitrile. A Picofrit Capillary column of 75 *μ*m I.D. × 7 cm with a 15 *μ*m spray tip packed with YMC ODSgel was conditioned for 10 minutes at 1 *μ*L/min with 100% solvent A before 5 *μ*L of the sample was injected and washed for 15 minutes at 1 *μ*L/min with 100% solvent A, followed by a gradient of 0 to 45% solvent B over 33 minutes and 2 minutes of 80% solvent B at a flow rate of 250 nL/min. The outlet of the column was placed inside a modified Michrom ADVANCE source coupled to the mass spectrometer's inlet with an ESI voltage of +1200 volts. Data acquisition consisted of one parent MS scan in the orbitrap from 350 to 2000 m/z with 60,000 resolution and 6 data-dependent MS/MS scans collected in the LTQ ion trap at 35 relative collision energy units. A charge state of +1 was rejected in the parent scan for analysis. 

### 2.5. Multiple Reaction Monitoring

Multiple reaction monitoring experiments of yeast enolase tryptic peptides were performed using a TSQ Vantage triple quadrupole mass spectrometer (Thermo Scientific) coupled to an Easy-nLCII capillary HPLC (Thermo Scientific). Tryptic digest of the yeast enolase standard was utilized for selection of the most intense and reproducible transitions through a few rounds of injections among the collection of transitions predicted from a theoretical digest of the protein, allowing one missed cleavage, using *Pinpoint* software (Thermo Scientific). Collision energies were optimized for each transition, resulting in a method monitoring 16 peptides using a total of 54 transitions. Analytical method details as well as the MS/MS transitions monitored are contained in the Supplementary Tables available online at http://dx.doi.org/10.1155/2013/674282.

### 2.6. Peptide Identification

Peptide identification from the data-dependent LC/MS experiment was conducted using a statistical wrapper to post-process the outputs of the *OMSSA *[14], *X! Tandem* [15], and *Protein Pilot* [16] software programs with a decoy database strategy of reversed protein sequences to limit false positive identifications. The approach is similar to the so-called “percolator” algorithm originally described by Käll et al. [17, 18], in which we used a random forest model to construct a classifier for correct peptide identifications using the search engine outputs in combination with other features such as delta mass, charge state, and so forth. Peptide identifications with *q*-values (false discovery rate estimates) less than 0.10 in a sample were retained for further analysis. Protein sequences from the UniProt Bovidae [19] family were combined with the sequences for yeast enolase and the 48 UPS1 proteins for all identifications. To maximize the coverage of proteins identified in a study, peptide identifications from all samples in the study were combined to create a list of peptides to quantify in each sample regardless of whether the particular peptide was identified in the sample. The rationale for this approach is that due to the random nature of triggering the data-dependent MS/MS scan, many peptides with low abundance fail to be identified in any given sample even though their concentrations are above the lowest detectable level in MS1, resulting in identification of the peptide in some but not all samples. By utilizing the combined list of peptides for quantification, a confident identification only needs to be made once out of any sample in order for the associated peptide ion current to be quantified in all study samples. This method significantly increased the number of quantifiable peptides and eliminated the problem of missing values commonly encountered in label-free MS1 quantification methods.

## 3. Results

### 3.1. Discovery Profiling—Quantification Algorithm

Relative quantification was accomplished by integrating the peak area of extracted ion chromatograms (XICs) from the primary (MS1) mass spectra. To fully leverage the specificity in high resolution MS data, XICs were constructed by summing together the ion current ±4 ppm around individual theoretical isotope peaks. In order to balance the increase in sensitivity by summing ion current from multiple isotopes with the chances for nonspecific interference by including more isotopes, only the ion currents from the minimum number of consecutive isotope peaks that gave at least 75% of the total ion current from the isotope profile were included. Selection of these isotope peaks was made by calculating the theoretical isotope distribution from the identified peptide sequence. Alternatively, in the absence of an identified peptide sequence or for computational speed considerations, we provide heuristics derived by surveying the computed isotope envelop of 32,100 peptides (Supplementary Table) that can be used to select the isotopes to include for XIC generation based only on the measured mass of the peptide. For example, a peptide with a monoisotopic molecular weight of 3700 Da would have an XIC generated by summing the ion current in 4 ppm m/z windows around the ^13^C isotope and the next heavier three isotopes.

Quantification of a protein began by assembling a list of all peptide ions identified in a study and the corresponding sample(s) in which the identifications were made. A peptide ion is defined by an amino acid sequence, charge state, and modifications. Since there can be multiple identifications of the same peptide ion within a sample (e.g., peptide elutes again at a high organic phase of the gradient), the retention time associated with the identification having the most intense MS/MS spectrum (as defined by the base peak in the MS/MS spectrum) was recorded. An extracted ion chromatogram for the peptide ion was then constructed and the centroid in a −0.25 min to +0.5 min retention time window around the MS/MS identification retention time was recorded for the peptide ion in the sample. The median of all centroid retention times for all samples containing the peptide ion identification was retained as the time at which to integrate the peptide ion. XICs around this median retention time are then generated and integrated for each sample in the study using the median retention time ±0.5 minutes irrespective of whether the particular peptide was identified in an individual sample. This method of consistently defined numerical integration of all peptides in each sample addressed one important frequently-encountered problem in MS1 quantification: missing data due to samples not containing an identification of a peptide. A local linear estimate for the baseline XIC ion current can optionally be subtracted prior to integration though we find this is generally not necessary for high resolution MS data. Peak picking of the XIC was not done, but rather, a straightforward numerical integration (trapezoid rule) was used to estimate the peak area within a defined retention time window. This approach requires reproducible chromatography so that the XIC peak is contained within the integrated retention time zone. For the results presented here, the nano-LC chromatography met this requirement. For cases when the chromatography is less reproducible, we have found chromatographic alignment as described in Higgs et al. has worked well as a pre-processing step to XIC integration [1].

### 3.2. Discovery Profiling—Protein Level Inference Model-Based (Concordant) Selection of Peptides

Three different methods for combining peptide level quantification information about a protein were evaluated: overall average of all peptide ions, average of model based selection of peptides, and the average of optimal dilution peptide selection. The overall average method simply averages the log_2_ transformed peptide ion peak areas for each peptide ion attributed to a protein. This method is simple but is adversely affected by including problematic peptide ions in the overall average. The model based peptide selection method accounts for the fact that not all peptide ions from a protein are informative for protein-level quantification. For example, peptides that are corrupted by background matrix, false positive identifications, or peptides from a low abundance post-translational modification will be discordant relative to peptides that do not suffer from interference or concentration limitations. The aim of model based peptide selection is to identify the largest set of concordant peptides from a protein and then average the log_2_ transformed peak areas from those selected peptide ions. Model based selection of peptides is based on fitting a simple linear model of the form

(1) $$\begin{array}{c} \log_{2}\left({\text{AUC}}_{ij}\right)=\mu +P_{i}+S_{j}+\varepsilon_{ij} \end{array}$$

in order to decompose the observed peptide ion peak areas for a protein into a peptide ion term *P*_*i*_ and a sample term *S*_*j*_, where AUC_*ij*_ is the peptide ion peak area for the *i*th peptide ion of the protein in the *j*th sample, *μ* is the overall mean (intercept), *P*_*i*_ is the effect attributed to the *i*th peptide ion (reflects different ionization efficiencies of peptides), *S*_*j*_ is the effect attributed to the *j*th sample (reflects varying levels of the protein in the study samples), and *e*_*ij*_ is the residual error. This additive model on log⁡_2_ transformed peak areas is motivated from the assumption that electrospray XIC peak areas are proportional to the product of a peptide ion specific constant and sample concentration of the peptide (AUC_*ij*_ ~ *P*_*i*_ *S*_*j*_). Peptide ions that are discordant with this linear model are candidates for exclusion (or down weighting) when estimating overall relative protein abundance from individual peptides. Discordance of peptide ions was estimated by examining the residuals from the regression described in (1). Discordant peptide ions will have residuals (*e*_*i*._) that have higher variance than the typical (median) peptide ion used in the regression. The proposed model based selection procedure is an iterative procedure described in the following 4 steps.

(1) For all peptide ions remaining in the data set for a protein, fit a linear model
  (2) $$\begin{array}{c} \log_{2}\left({\text{AUC}}_{ij}\right)=\mu +P_{i}+S_{j}+\varepsilon_{ij}. \end{array}$$
(2) Calculate the sample variance of the residuals for each peptide ion: Var(*e*_*i*._).
(3) Using a standard chi-squared test, compare the peptide ion with the highest residual variance to twice the median of the residual variance of all peptide ions fit in the model.
(4) If the test in step (3) is significant (*P* < 0.05), then remove the highest residual variance peptide ion from the data set and go to step (1), otherwise go to step (5).
(5) Repeat steps (1)–(4) for each protein identified in the study.

Additionally, a minimum and maximum number of peptide ions retained from this algorithm can be enforced, irrespective of the chi-squared test, by amending the stopping rule in step (4). For the work presented here we have adopted a simple, hard weighting, scheme where individual peptides are either retained or not for averaging (0/1 weighting). A maximum of the 20 most concordant peptide ions were retained for averaging in lieu of a more complex weighting scheme for peptides meeting the chi-squared test criterion. It is a natural extension of this work to perform a weighted average of the individual quantitative values for a peptide but that was not pursued at this time. 

### 3.3. Identification of Dilution Optimal Peptides

While the model based peptide ion selection is intended to identify the largest subset of concordant peptide ions from a protein, we were also interested in a systematic method to assess the existence of peptide ions that were optimal given a serial dilution. To accomplish this, we used a similar regression technique to identify a dilution optimal set of peptide ions. A regression model of the form log⁡_2_(AUC_*j*_) = *μ* + log⁡_2_(*C*_*j*_) + *e*_*j*_ was fit for each peptide ion associated with a protein, where AUC_j_ is the peptide ion peak area for the *j*th sample, *μ* is the intercept, *C*_*j*_ is the amount of analyte spiked into the sample, and *e*_*j*_ is the residual error. Peptide ions with a high coefficient of determination (*R* ^2^) value were selected as being dilution optimal. For the results presented here, peptide ions with *R* ^2^ values greater than 0.85 were defined as dilution optimal. If more than 20 peptide ions resulted in *R* ^2^ > 0.85 then the peptide ions corresponding to the twenty highest *R* ^2^ measures were retained. All statistical analyses were conducted in the *R* statistical computing environment [20].

As a first step in evaluating the proposed methods, fifteen replicate injections of the sixlog mix were evaluated on an LTQ-Orbi/Velos and an LTQ-Orbi/Elite mass spectrometer. Coefficients of variation (CV) values were estimated using the log_2_ transformed peak areas using the identity

(3) $$\begin{array}{c} \text{CV}\left(\%\right)=100\sqrt{e^{{\left(\ln(2)\hat{\sigma }\right)}^{2}}-1}, \end{array}$$

where $\hat{\sigma }$ is the sample standard deviation calculated from the 15 replicates. The median CV of all detected peptide ions for a protein along with the CV from taking a mean of all peptide ion log_2_ AUCs and a mean of all model selected (concordant) peptide ions indicate that the model based selection of peptide results in substantially lower CVs for the proteins in the sixlog mix (Table 1). This reduction in technical variation is significant with CVs generally twofold lower for the model selected peptides and not surprising given the nature of the iterative method to identify the most concordant (least variable) peptides.

**Figure 1:**
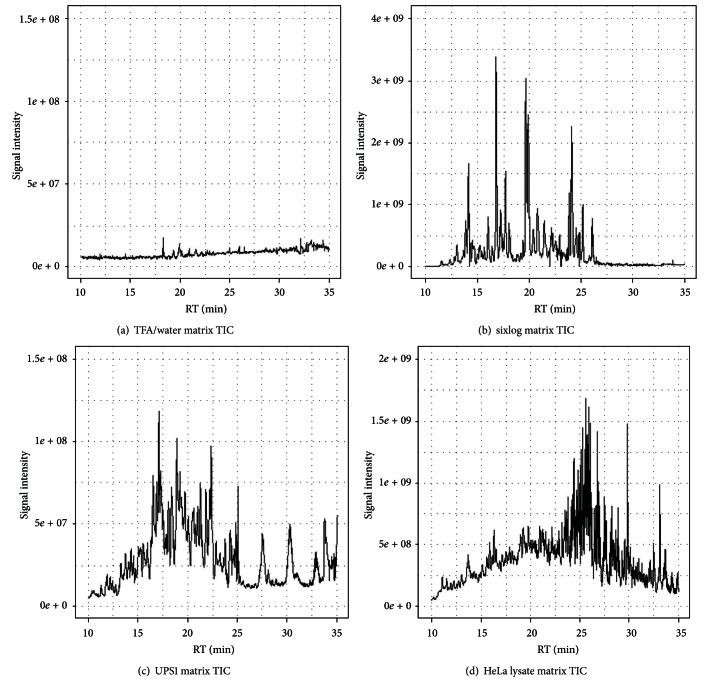
Total ion MS1 chromatograms for the background matrices used for yeast enolase dilutions. TFA/water (a), sixlog mix (b), UPS1 proteomics standard (c), and HeLa cell lysate (d).

**Figure 2:**
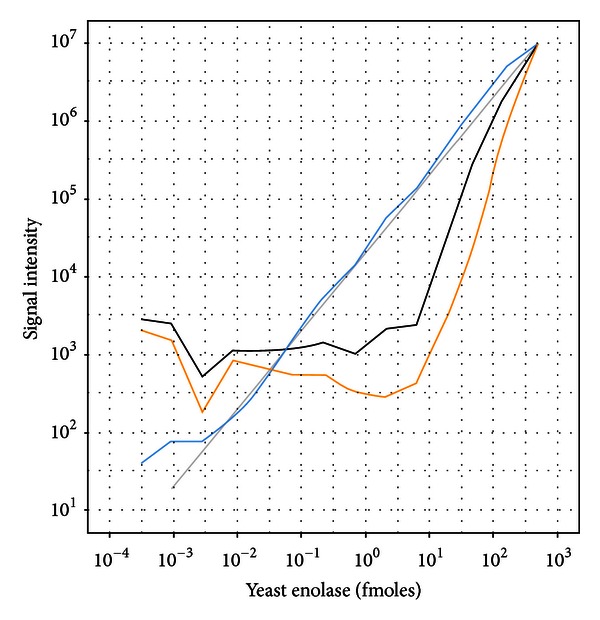
Dilutional linearity of yeast enolase titrated into a TFA/water background. Enolase quantification using the average of all peptides (black), average of model selected peptides (orange), and average of dilution optimal selected peptides (blue). Deviation from the theoretical dilution line for the model selected and all peptide curves is attributed to nonspecific binding with a TFA/water matrix.

In order to characterize the relative quantification performance in different background matrices, fourteen 3x dilutions of yeast enolase from 500 fmoles down to 313.6 zmoles were spiked into matrices consisting of TFA/water, sixlog mix, UPS1 proteomics standard, and a trypsin digest of a HeLa cell lysate (see Supplementary Tables for the enolase concentrations used). These matrices represent progressively more complex backgrounds that can interfere with or suppress the spiked enolase (Figure 1). The dilution curves for TFA/water using all peptides and using the model selected (concordant) peptides indicate dilution curves with slopes steeper than the spiked concentrations (Figure 2). This is presumably due to nonspecific binding (losses) of the peptides due to the lack of any other proteins in the matrix. The model selected (concordant) peptide average was not substantially different than just using the mean of all peptides. In order to determine if there were any peptides detected in the study that did not suffer from the nonspecific binding in the TFA/water matrix, we used the dilution optimal model selection to identify twenty peptides that fit the known dilution series with an *R* ^2^ greater than 0.85 (Figure 2, blue line). Presumably these peptides that are consistent with the known dilution of yeast enolase are more hydrophilic and less likely to bind to plastic and other surfaces with this simple background matrix. Indeed that was what was observed as the highest *R* ^2^ peptides were associated with the most hydrophilic peptides detected in the study (Supplementary Figure).

**Figure 3:**
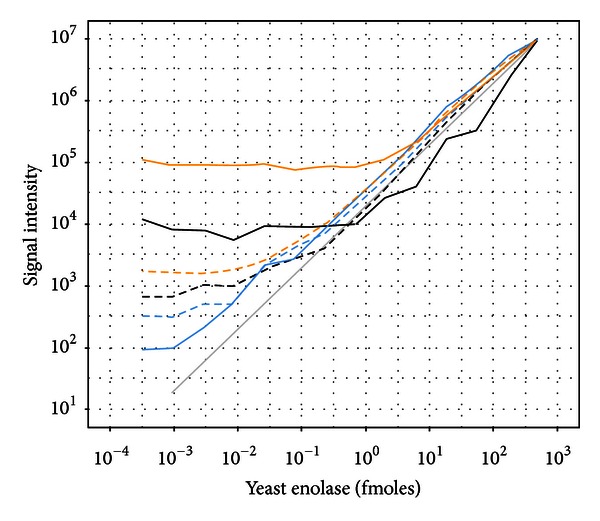
Dilutional linearity of yeast enolase titrated into a sixlog background. Global profiling results shown in solid lines and MRM results shown in dashed lines. Enolase quantification using the average of all peptides (black), average of model selected peptides (orange), and average of dilution optimal selected peptides (blue).

Next, a background matrix derived from a 500 fmol maximum concentration of the sixlog mixture was used for the yeast enolase dilution. In addition to the discovery mode MS1 quantification, these samples were also analyzed in an MRM mode to better understand the relative performance between these approaches (Figure 3). The average of the model based (concordant) peptides was not substantially different than just taking the average of all peptides, with quantification feasible from approximately 1 fmole and higher. However, using the dilution optimal model to screen for peptides that follow the known dilutions, we found that a number of peptides were identified that follow the known concentrations down to approximately 1 amole. For the MRM data, the average of all transition peak areas as well as the model based (concordant) and dilution optimal transitions performed similarly to each other and provided superior sensitivity relative to the average of all MS1 peptide quantifications and the model selected (concordant) MS1 peptides.

**Figure 4:**
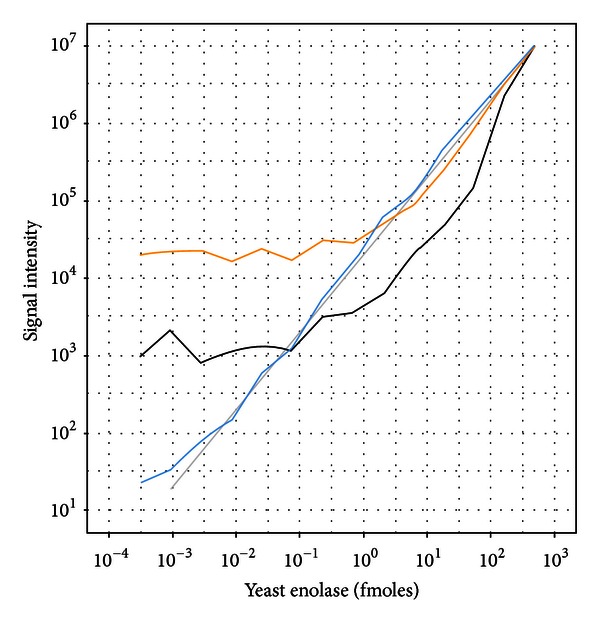
Dilutional linearity of yeast enolase titrated into a UPS1 background. Enolase quantification using the average of all peptides (black), average of model selected peptides (orange), and average of dilution optimal selected peptides (blue).

Titration of yeast enolase into a matrix comprised of 48 equimolar human proteins was conducted to complement the sixlog mix matrix as a more complex background that may be found from less specific enrichment protocols. The MS1 quantification results were similar to the sixlog mix matrix with the average of all peptides and the average of the model selected (concordant) peptides showing quantitative signal responses from 1 fmole and higher (Figure 4). Again, there were a number [20] of MS1 quantified peptides that were identified using the dilution optimal model that showed quantitative performance down to approximately 1 amole. 

**Figure 5:**
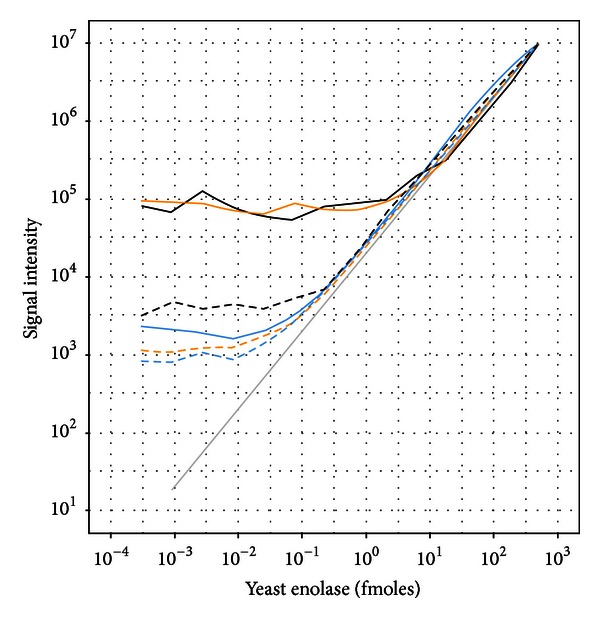
Dilutional linearity of yeast enolase titrated into a HeLa lysate background. Global profiling results shown in solid lines and MRM results shown in dashed lines. Enolase quantification using the average of all peptides (black), average of model selected peptides (orange), and average of dilution optimal selected peptides (blue).

Lastly, yeast enolase was diluted into a highly complex HeLa cell lysate digest (Figure 5). The average of all MS1 quantified peptides performed similarly to the model selected (concordant) peptides. With this more complex matrix the MRM quantifications outperformed the MS1 quantification counterparts for the average of all peptides as well as the average of model selected (concordant) peptides. Interestingly, there were still a number of MS1 quantified peptides that performed as well as the best set of MRM transitions with quantification possible down to concentrations around 25 amoles. The association between peptide hydrophobicity and dilution optimal model R2 was not observed for the HeLa matrix as it was for the TFA/water matrix (Supplementary Figure). This is likely due to the fact that high R^2^dilution optimal peptides derive from MS1 m/z values that are not contaminated with any interfering signals from the background HeLa matrix which should not necessarily show any association with hydrophobicity of the peptides. 

## 4. Discussion

This report serves as an extension to our previously described approach [1] to label-free relative peptide and protein quantification for discovery, hypothesis generating, experiments using previous generation of nominal mass accuracy hardware. This extension includes the following aspects which we have found to have significant improvement over the first generation of label free quantification.

1. Since quantification is performed using an overall list of peptides identified from any sample in a study, if possible, it is advantageous to include a sample containing the target protein(s) at a high concentration in order to “seed” the analysis to quantify a maximum number of peptides. Many peptides can be quantified in a sample from their MS1 signal even if an MS2 event was never triggered in that particular sample due to low abundance, eliminating the problem of how to handle missing data in the earlier generation experiments.
2. The high mass accuracy and resolution possible with current generation mass spectrometers has dramatically increased the specificity of quantification using MS1 signals. In fact, for all of our different background matrices we were able to identify numerous peptides with MS1 signals unaffected by matrix ions that performed as well as MRM transitions using a triple quadrupole instrument. Identifying the smallest number of isotope peaks accounting for a majority (>75%) of the signal in the isotope distribution provides a good balance between increasing sensitivity while limiting exposure to interfering signals from the matrix. We also provide a heuristic table (Supplementary Tables) that can guide selection of the isotopes for those who do not have an easy access to calculation of theoretical isotope envelope.
3. We provide improved methods to estimate the elution time of a peptide for quantification. Often times there are multiple data-dependent MS2 scans of the same peptide ion within a sample. MS1 quantification relies on a good estimate of the retention time of the peptide ion. We have found that using the MS2 scan time corresponding to the maximum MS2 fragment ion intensity provides a reliable estimate for the retention time of the maximum peptide ion signal within a sample. Using a robust estimator like the median of the peptide ion retention times for a peptide ion across multiple samples in a study generally produces high quality estimates for peptide ion quantification.

Using these improvements, we evaluated multiple methods to improve measurement of protein level from measurements of multiple peptides derived from the same protein. While we certainly advocate performing hypothesis tests at both the peptide ion level as well as the protein level, the optimal approach to protein level inference from multiple peptide quantifications was not apparent. Acknowledging that MS1 quantification may be more susceptible to background matrix interference than an MRM method, we were motivated to identify a simple, iterative algorithm using a linear model to find the largest set of concordant peptides within a study. The rationale for this investigation was that an average using this concordant set of peptides would represent an optimal estimate for the relative levels of the protein in the samples. Overall we found this method worked well at reducing technical variability for the protein but, by design, could not correct for situations where the majority of peptides were affected by interference or ion suppression. Interestingly, we were able to identify at least twenty peptide ions in each of our four matrices that provided similar quality quantitative responses as the best MRM transitions. This observation motivates an alternative view to relative protein quantification using MS1 signals. Specifically, our results suggest that it is optimal to employ meta-analysis techniques to summarize the inference results from the peptides rather than combining the peptide quantifications and then performing the protein level inference [21, 22]. For example, consider a study in which a protein of interest is in the 0.1–1 fmole range in a background similar to a HeLa lysate (Figure 5). Using an average of all peptides or the model selected (concordant) peptides in this example would not enable detection of different protein levels between groups due to the flat instrument response in this concentration range (Figure 5, solid black and orange curves). However, we know that a number of peptides exist in this concentration range that would allow the discovery of differential protein levels (Figure 5, solid blue curve). A meta-analysis of the peptide ion inference results would likely be the most powerful test of the protein in this example as we clearly showed the existence of a number of peptides that showed a linear response well below the quantification limits from the average of all peptides or concordant peptides. That is, with the knowledge that low level proteins can have peptides with useful quantitative information, the existence of multiple peptides from a protein showing similar magnitude, direction, and statistical significance would warrant further investigation into the protein. Alternatively, performing a standard addition analysis using the protein of interest and the relevant matrix would enable the use of the dilution optimal model as a means to identify an optimal set of peptides for the highest sensitivity. While the model-based (concordant) peptide selection approach turned out to be not very useful in the MS1 quantification, we found that this method may be most useful for post-processing a set of MRM transitions for a protein. Similar to MS1 quantification, the question of how an investigator should combine the information from multiple MRM transitions for protein level inference needs to be addressed, particularly when these transitions can be corrupted by interfering matrix signals, differences in sensitivity, and so forth. Arithmetic mean, geometric mean, averaging transitions to peptides and then peptides to the protein level, as well as linear mixed models have been proposed [13]. To our knowledge, none of these methods provide an objective, and automated mechanism for eliminating problematic transitions. If a dilution series of the protein of interest into a relevant matrix devoid of the protein of interest is available, then the dilution optimal model selection described here provides a simple, yet powerful, approach to objectively identify a small set of transitions to monitor. In the absence of a dilution series, the model based (concordant) selection method can be used to select MRM transitions. Our results indicate that, in both simple and complex backgrounds, both approaches yield similar results for identifying a quality set of MRM transitions.

We have reported here an MS1 relative quantification method that leverages the high mass accuracy and resolution of modern mass spectrometers, approaches to post-processing individual peptide ion peak areas, and a comparison of this approach to targeted MRMs in four different background matrices. Overall, we have found significant improvements over the first generation of MS1 relative quantification approaches with sensitivity and specificity results approaching that of targeted MRM methods. These improvements in data analysis methodologies in combination with the new generation high mass accuracy hardware should benefit researchers working in multiple hypothesis-generating settings such as chemiproteomics, immunoprecipitation mass spectrometry, protein-protein interaction mapping, and profiling of specific classes of proteins (e.g., acylated, phosphorylated, etc.).

## Supplementary Material

Table S1: Instrument settings used for the multiple reaction monitoring (MRM) experiments.
Table S2: Parent and daughter ions used to quantify yeast enolase with the MRM experiments.
Table S3: Yeast enolase concentrations used in all experiments.
Table S4: Lookup table that can be used to identify the smallest number of isotopes in an isotope distribution in order to retain *∼*75% of the total signal in the isotope distribution.
Figure S1: Retention time vs. dilutional optimal model R2 value demonstrating the effects of non-specific binding with a TFA/water background matrix.

## Figures and Tables

**Table 1:** Reproducibility estimates from fifteen repeated injections of sixlog mix. Top row are results from an LTQ-Orbi/Velos and bottom row are results from an identical experiment conducted on an LTQ-Orbi/Elite. Peptide selections were done using the model based approach. While *α*-casein is reported here we question the absolute amount in the sixlog mix (50 amol) based on the large number of peptides identified and the possibility of copurification from the more abundant components.

Protein	Number selected/ number of total peptides	Median peptide CV (%)	Mean of all peptides CV (%)	Mean of model based selected peptides CV (%)
*β*-Lactoglobulin (500 fmol)	20/725	42.7	23.4	4.9
	20/1033	19.0	8.2	3.1
Lactoperoxidase (50 fmol)	20/248	21.2	13.9	4.2
	20/482	21.2	9.6	4.0
Carbonic anhydrase (5 fmol)	20/49	15.6	16.1	5.5
	20/84	20.2	12.3	3.4
Glutamate dehydrogenase (500 amol)	11/27	42.1	29.1	11.3
	6/26	50.3	63.5	3.4
*α*-casein (50 amol)	17/32	26.4	22.7	10.4
	29/42	18.7	13.5	7.3
