# Supplementary material for: Quantitative Proteomics via High Resolution MS Quantification: Capabilities and Limitations
Source: Int J Proteomics. 2013 Apr 23;2013:674282. doi: 10.1155/2013/674282 (PMC3655581; doi:10.1155/2013/674282)
Supplement: Supplementary file 1 — Table S1: Instrument settings used for the multiple reaction monitoring (MRM) experiments. Table S2: Parent and daughter ions used to quantify yeast enolase with the MRM experiments. Table S3: Yeast enolase concentrations used in all experiments. Table S4: Lookup table that can be used to identify the smallest number of isotopes in an isotope distribution in order to retain ∼75% of the total signal in the isotope distribution. Figure S1: Retention time vs. dilutional optimal model R2 value demonstrating the effects of non-specific binding with a TFA/water background matrix. [file 674282.f1.docx]

**Supplementary Tables and Figures**

**Table S1: MRM mass spectrometry method**

Mass Spec: **Thermo Scientific Inc. (Waltham, MA)**

TSQ Vantage Triple Stage Quadrupole Mass Spectrometer

Liquid Chromatography: **Thermo Scientific Inc. (Waltham, MA)**

Easy-nLCII

LC Buffer A: **Thermo Scientific Inc. (Waltham, MA)**

0.1% Formic Acid in Water (v/v) Solvent Blends, Optima® LC/MS ≥99.0%

LC Buffer B: **Thermo Scientific Inc. (Waltham, MA)**

0.1% Formic Acid in Acetonitrile (v/v) Solvent Blends, Optima® LC/MS 40 to 42%

Capillary Column: **New Objective, Inc. (Woburn, MA)**

75micron x 70mm Capillary Column YMC GEL ODS

SRM Selection Software: **Thermo Scientific Inc. (Waltham, MA)**

Pinpoint Version 1.1.0

LCMS Standard 6log: **MICHROM Bioresources, Inc. (Auburn, CA)**

6 Bovine Tryptic Digest Exponential Molar Mix (P/N PTD/00001/64)

LCMS Standard Enolase: **MICHROM Bioresources, Inc. (Auburn, CA)**

Yeast Enolase (PTD/00001/46)

Instrument Parameters:

Tune Method:

Capillary Temperature: 200.0

Vaporizer Temperature: 330.0

Sheath Gas Pressure: 0.0

Ion Sweep Gas Pressure: 0.0

Aux Valve Flow: 0.0

Spray Voltage: Positive polarity - 1200.0, Negative polarity - 3000.0

Discharge Current: Positive polarity - 4.0, Negative polarity - 4.0

TSQ Method Settings:

Method Type: EZ Method

MS Run Time (min): 35.0

Experiment Type: SRM

Chrom Filter Peak Width (s): Not used

Collision Gas Pressure (mTorr): 1.0

Use Tuned S-Lens Value: Yes

Q1 Peak Width (FWHM): 0.70

Display Time Range for SRM table: Yes

Cycle Time (S): 1.000

DCV (V): Not used

Thermo Easy-nLC :

Sample pickup:

Volume (ul): 5.00

Flow (ul/min): 20.00

Sample loading:

Volume (ul): 15.00

Flow(ul/min): 1.0

Max. Pressure (Bar): 280.00

Gradient:

Time (mm:ss) Duration (mm:ss) Flow (nl/min) Mixture (%B)

00:00 00:00 250.00 2.00

32:00 32:00 250.00 45.00

33:00 1:00 250.00 80.00

35:00 2:00 250.00 80.00

Analytical Column equilibration:

Volume (ul): 10.00

Flow (ul/min): 1.0

Max. Pressure (Bar): 280.00

**Table S2: Parent and daughter MRM ions**

| **Sequence** | **Parent Ion m/z** | **Daughter Ion m/z** |
| --- | --- | --- |
| NVNDVIAPAFVK | 643.859 | 561.32 |
| NVNDVIAPAFVK | 643.859 | 632.32 |
| NVNDVIAPAFVK | 643.859 | 745.42 |
| NVNDVIAPAFVK | 643.859 | 1073.52 |
| AVDDFLISLDGTANK | 789.904 | 605.225 |
| AVDDFLISLDGTANK | 789.904 | 805.425 |
| AVDDFLISLDGTANK | 789.904 | 918.425 |
| AVDDFLISLDGTANK | 789.904 | 1031.525 |
| TFAEALR | 404.222 | 488.215 |
| TFAEALR | 404.222 | 559.315 |
| IGSEVYHNLK | 580.309 | 511.224 |
| IGSEVYHNLK | 580.309 | 674.324 |
| IGSEVYHNLK | 580.309 | 773.424 |
| IGSEVYHNLK | 580.309 | 989.524 |
| IGLDC[Carboxymethyl]ASSEFFK | 687.816 | 744.327 |
| IGLDC[Carboxymethyl]ASSEFFK | 687.816 | 815.327 |
| IGLDC[Carboxymethyl]ASSEFFK | 687.816 | 976.427 |
| IGLDC[Carboxymethyl]ASSEFFK | 687.816 | 1091.427 |
| DGKYDLDFK | 550.767 | 409.226 |
| DGKYDLDFK | 550.767 | 522.223 |
| DGKYDLDFK | 550.767 | 637.323 |
| DGKYDLDFK | 550.767 | 800.323 |
| YDLDFK | 400.695 | 409.217 |
| YDLDFK | 400.695 | 522.214 |
| YDLDFKNPNSDK | 728.341 | 560.232 |
| YDLDFKNPNSDK | 728.341 | 674.332 |
| YDLDFKNPNSDK | 728.341 | 802.429 |
| YDLDFKNPNSDK | 728.341 | 949.432 |
| TAGIQIVADDLTVTNPK | 878.478 | 1002.531 |
| TAGIQIVADDLTVTNPK | 878.478 | 1073.528 |
| TAGIQIVADDLTVTNPK | 878.478 | 1172.631 |
| TAGIQIVADDLTVTNPKR | 956.529 | 1158.639 |
| TAGIQIVADDLTVTNPKR | 956.529 | 1229.639 |
| TAGIQIVADDLTVTNPKR | 956.529 | 1328.739 |
| RIATAIEK | 451.277 | 460.222 |
| RIATAIEK | 451.277 | 561.319 |
| RIATAIEK | 451.277 | 632.319 |
| KAADALLLK | 471.803 | 557.42 |
| KAADALLLK | 471.803 | 672.42 |
| KAADALLLK | 471.803 | 743.42 |
| AADALLLK | 407.755 | 486.315 |
| AADALLLK | 407.755 | 557.415 |
| AADALLLK | 407.755 | 672.412 |
| VNQIGTLSESIK | 644.859 | 563.326 |
| VNQIGTLSESIK | 644.859 | 834.426 |
| VNQIGTLSESIK | 644.859 | 947.523 |
| SGETEDTFIADLVVGLR | 911.465 | 771.435 |
| SGETEDTFIADLVVGLR | 911.465 | 842.532 |
| SGETEDTFIADLVVGLR | 911.465 | 955.535 |
| SGETEDTFIADLVVGLR | 911.465 | 1102.635 |
| LAKLNQLLR | 534.848 | 401.222 |
| LAKLNQLLR | 534.848 | 529.322 |
| LAKLNQLLR | 534.848 | 643.325 |
| LAKLNQLLR | 534.848 | 756.416 |

**Table S3: Yyeast enolase dilutions**

| sample | fmoles | amoles | zmoles |
| --- | --- | --- | --- |
| working stock | 15000 |  |  |
| 0 | 1500 |  |  |
| 1 | 500.000 | 500000.000 | 500000000.000 |
| 2 | 166.667 | 166666.667 | 166666666.667 |
| 3 | 55.556 | 55555.556 | 55555555.556 |
| 4 | 18.519 | 18518.519 | 18518518.519 |
| 5 | 6.173 | 6172.840 | 6172839.506 |
| 6 | 2.058 | 2057.613 | 2057613.169 |
| 7 | 0.686 | 685.871 | 685871.056 |
| 8 | 0.229 | 228.624 | 228623.685 |
| 9 | 0.076 | 76.208 | 76207.895 |
| 10 | 0.025 | 25.403 | 25402.632 |
| 11 | 0.008 | 8.468 | 8467.544 |
| 12 | 0.003 | 2.823 | 2822.515 |
| 13 | 0.001 | 0.941 | 940.838 |
| 14 | 0.000 | 0.314 | 313.613 |

| **Mono MW** | **Starting**  **Isotope** | **Number of**  **Isotopes** |
| --- | --- | --- |
| < 500 | 1 | 1 |
| 500-1,430 | 1 | 2 |
| 1,430-2,540 | 1 | 3 |
| 2,540-3,600 | 1 | 4 |
| 3,600-4,350 | 2 | 4 |
| 4,350-5,900 | 2 | 5 |
| 5,900-6,600 | 3 | 5 |
| 6,600-8,250 | 3 | 6 |
| 8,250-9,200 | 4 | 6 |
| 9,200-10,450 | 4 | 7 |
| >10,450 | 5 | 7 |

**Table S4: Lookup table for determining which isotopes to include in XIC generation. Monoisotopic (^12^C) isotope is designated as isotope 1, ^13^C isotope is designated as isotope 2, etc. Selection of isotopes using this heuristic will generally provide 75% or more of the total signal in the isotope envelope with the minimum number of consecutive isotope peaks.**


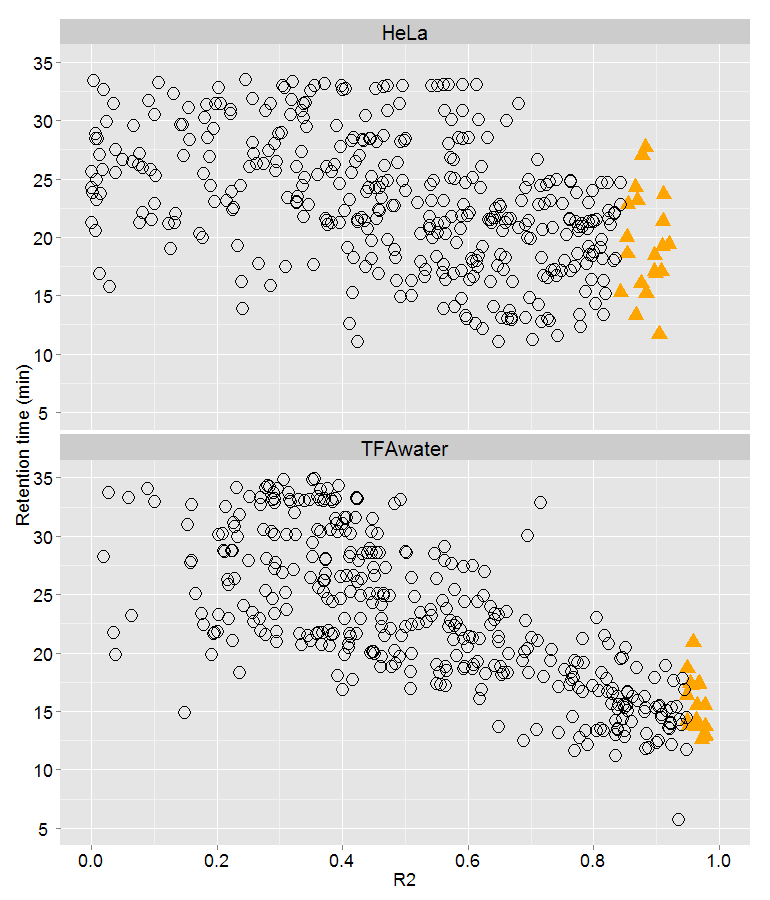


**Figure S1: Peptide retention time vs. dilutional linearity model fit R^2^ for the HeLa matrix (top panel) and the TFA/water matrix (bottom panel). Orange triangles designate the highest twenty R^2^ peptides for each matrix. Note the trend for earlier retention time peptides (more hydrophilic) for the higher R^2^ (better fitting) peptides to the dilution model for the TFA/water matrix.**
